# Constitution

**Published:** 1852-04

**Authors:** 


### CONSTITUTION.

Article i. This society shall be called the Ohio Dental College Association.

Article ii. The officers of this Association shall consist of a President, two Vice Presidents, a Secretary, a Treasurer, and an Examining Committee of five; three from the dental, and two from the medical profession, who shall be chosen by ballot at each Annual Meeting of the Association, and who shall perform such duties as usually pertain to their respective offices.

Article iii. The members of this Association shall consist of two classes: 1. The holders of stock in the Ohio Dental College; 2. All graduates of the institution may become members on receiving a vote of two-thirds of the members present, signing the constitution, and obligating themselves to pay annually into the treasury a sum equal to the interest on one share of stock.

Article iv. Any member may be expelled by a vote of two-thirds of the members present, for immoral or unprofessional conduct.

*Section* 1. No expelled member shall have any of his annual contributions refunded, but if he is a stockholder, he may sell his stock, always giving the Association the first privilege as purchaser.

*Section* 2. Stock may be sold or tranferred, but the purchaser shall not be entitled to membership except by a vote of two-thirds of the members present at any annual meeting.

*Section* 3. The purchase of a share of stock shall not entitle the holder to membership unless he shall receive a vote of two-thirds of the members present.

Article v. The meetings of the Association shall be held annually, in the Ohio Dental College in Cincinnati, at 10 o'clock A. M. of the day preceeding the annual commencement; and the President may call a meeting when requested by five members; and in all meetings it shall require thirteen to form a quorum.

Article vi. In all matters relating to the property held by the Association, each stockholder shall have as many votes as he may have shares of stock, and in case of unavoidable absence he may vote by proxy.

Article vii. This constitution may be altered or amended by a vote of two-thirds present at any Annual Meeting, except such change as would affect the shares of stock; which amendment must be proposed at one Annual Meeting, and acted on at the next.

After the adoption of the constitution the Association went into an election for officers, which resulted as follows:

DR. JAMES TAYLOR, President.
" W. M. WRIGHT, 1st Vice President.
" THOS. WOOD, 2nd Vice President.
" CHAS. BONSALL, Secretary.
" EDWD. TAYLOR, Treasurer.

EXAMINING COMMITTEE.

DR. SOMERBY, of Louisville, Ky., } Dental.

" HALE, of St. Louis, Mo., } Dental.

" TALBERT, of Dayton, Ohio, } Dental.

" P. S. CONNER, of Cincinnati, } Medical.

" P. G. FORE, of Cincinnati, } Medical.

After getting through the election, the following gentlemen, alumni of the college, were proposed for membership, and were unanimously elected:

DR. W. H. GODDARD of Louisville, Ky.,
" SAM. GRIFFITH, of Louisville, Ky.,
" GEO. L. PAINE, of Xenia, Ohio,
" J. A. HERRING, of Kosciusko, Miss.

Adjourned to meet in the College at 9 A. M. to-morrow.

MORNING SESSION.

Met according to adjournment, at 9 A. M. of the 20th.

The minutes of yesterday were read and adopted.

On motion of Dr. Ulrey, the Treasurer was directed to pay the bill presented to-day for examining title, making abstract, &c., of the college property.

On motion of Dr. Goddard, *Resolved*, That the members be requested to use exertions to add to the library and cabinet of the College.

On motion of Dr. Manlove, *Resolved*, That the stockholders hereby allow all the moneys accruing from interest on stock, admission of members, and other sources, to be appropriated for the next three years for repairs and improvement to the college building.

On motion of Dr. Smith, *Resolved*, That Drs. James Taylor, Wood and Allen be a committee to inquire and report plans and cost for the improvement of the building at the next Annual Meeting.

On motion, adjourned to meet at 3 P. M.

AFTERNOON SESSION.

The morning's minutes were read and approved, after which,

On motion of Dr. Griffith, *Resolved*, That the President appoint a committee of three to prepare a code of bye-laws, and report at next meeting. He appointed Drs. Mendenhall, Bonsall and Van Emon.

The following was received from the faculty of the institution: *Resolved*, That thanks of the faculty be offered to the members of the profession for the material aid they have so generously given in the cause of the college; after which,

On motion of Dr. Smith, *Resolved*, That the stockholders approve of the present organization of the Dental College.

On motion of Dr. Bonsall, *Resolved*, That the Treasurer procure a book for himself, and one for the use of the Secretary.

On motion of Dr. Wood, *Resolved*, That the President be authorized to send invitations to such members of the dental and medical professions as he may see proper, to be present at the annual examination and commencement.

On motion of Dr. Wright, *Resolved*, that the proceedings of this association at its present meeting, be published in the Dental Register of the West.

Two members were appointed to prepare essays on the following subjects for next Annual Meeting, viz., Dr. Geo. Mendenhall, on Professional Etiquette, and Dr. J. Taft, on Dental Jurisprudence.

The members present now signed the constitution, as follows:

Wm. H. Goddard, *Louisville, Ky.*,

Sam. Griffith, " "

James Taylor, *Cincinnati, O.*,

Charles Bonsall, " "

W. H. Wright, *Pittsburg, Pa.*,

J. P. Ulrey, *Lawrenceburg, Ind.*,

J. Taft, *Xenia, O.*,

Geo. L. Paine, *Xenia, O.*,

H. R. Smith, *Terre Haute, Ind.*,

G. L. Van Emon, *Cincinnati, O.*,

Jno. Allen, " "

W. C. Duncan, " "

Edwd. Taylor, " "

M. N. Manlove, *Logansport, Ind.*,

Geo. Mendenhall, *Cincinnati, O.*,

Thos. Wood, " "

A. S. Talbert, *Dayton, O.*,

J. A. Herring, *Kosciusko, Miss.*

The following stockholders were represented by proxy:

Dr. Sam. B. Fithian, *St. Louis, Mo.*,
" C. W. Spalding, " "
Messrs. Jones, White & Co., *Philadelphia, Pa.*,
Dr. A. Berry, *Raymond, Miss.*,
" W. S. Chandler, *Port Gibson, Miss.*,
" H. E. Peebles, *Lexington, Mo.*,
" John S. Clark, *New Orleans, La.*,
" James S. Knapp, " "
" Daniel Dougherty, *Bardstown, Ky.*,
Mr. J. L. Talbott, *Cincinnati, O.*,
" J. B. Dunlevy, *Pittsburg, Pa.*,

After which the association adjourned *sine die.*

Signed, CHAS. BONSALL, SECRETARY.

